# Report of a Meeting on Contemporary Topics in Zebrafish Husbandry and Care

**DOI:** 10.1089/zeb.2016.1324

**Published:** 2016-12-01

**Authors:** Nikki Osborne, Gregory Paull, Adam Grierson, Karen Dunford, Elisabeth M. Busch-Nentwich, Lynne U. Sneddon, Natalie Wren, Joe Higgins, Penny Hawkins

**Affiliations:** ^1^ Research Animals Department, RSPCA, Southwater, United Kingdom.; ^2^ University of Exeter, Exeter, United Kingdom.; ^3^ University College London, London, United Kingdom.; ^4^ Wellcome Trust Sanger Institute, Cambridge, United Kingdom.; ^5^ University of Liverpool, Liverpool, United Kingdom.; ^6^ Francis Crick Institute (previously National Institute for Medical Research), London, United Kingdom.

**Keywords:** animal welfare, refinement, Three Rs, environmental enrichment, husbandry, colony management

## Abstract

A meeting on *Contemporary Topics in Zebrafish Husbandry and Care* was held in the United Kingdom in 2014, with the aim of providing a discussion forum for researchers, animal technologists, and veterinarians from academia and industry to share good practice and exchange ideas. Presentation topics included protocols for optimal larval rearing, implementing the 3Rs (replacement, reduction, and refinement) in large-scale colony management, and environmental enrichment. The audience also participated in a survey of current practice relating to practical husbandry, cryopreservation, and the provision of enrichment.

## Introduction

The RSPCA Transgenic Training Working Group (TTWG) was set up in October 2006 in response to several forums that had identified a need for specialist training in the implementation of the 3Rs (replacement, reduction, and refinement) in relation to procedures involving the creation, breeding, care, and use of transgenic animals.^1–4^ The group also turned its attention to the care and use of zebrafish because of the ease with which this species can now be genetically altered, and the significant recent annual increases in the number of zebrafish used in research and testing.

For example, there were 194,562 procedures using “fish” (including zebrafish) in 2004,^5^ which has increased to 285,697 procedures using zebrafish alone in 2014.^6^ This reflects an increase in zebrafish use in both basic and applied biomedical research, and over 150,000 of these procedures relate to the creation and breeding of GA strains.

Despite this increase in use, there are few standard husbandry protocols between groups and facilities in the United Kingdom or worldwide.^7^ Standard good husbandry practices will only become commonplace through improved understanding, and communication between users, of the behavior and life-history traits of zebrafish, which need to be underpinned by sound animal welfare science. More attention seems to have been paid to the genetics and health status of zebrafish, with researchers keen to establish standardized background strains that have well-defined characteristics, and work has also begun in earnest to scrutinize the health status of zebrafish, including the creation of specific pathogen-free fish.

All of the above is already common practice for laboratory rodents, and it is increasingly recognized that the same scientific health and animal welfare considerations should apply to zebrafish. For example, an ongoing joint Federation of European Laboratory Animal Science Associations (FELASA) and COST (European Cooperation in Science and Technology) action on *Zebra fish: housing, husbandry, and health monitoring recommendations* is reviewing available information and will issue guidelines on basic housing and husbandry practices for zebra fish that promote the animals' health and well-being.^8^

The RSPCA TTWG meeting on *Contemporary topics in zebrafish husbandry and care* therefore aimed to bring animal technologists, veterinarians, and scientists together to discuss approaches to monitoring zebrafish health and welfare, exchange ideas, and disseminate good practice. Summaries of the presentations and discussion are set out below.

## Larval Rearing: Overview of Various Methods Within Zebrafish Facilities

Karen Dunford (University College London [UCL]) provided an overview of different larval rearing methods at zebrafish facilities both within the United Kingdom and worldwide. She began by discussing good husbandry at various stages of development, starting with the rapid growth that is typical of morphogenesis (0–3 dpf). Embryos are typically housed in Petri dishes in incubators, free from dead eggs and debris, but stocking densities in the literature vary from 16 to 1000 embryos per dish. This may partly reflect variations in Petri dish size, but at UCL, it has been found that stocking embryos at too high a density (200+ per dish) will result in depleted oxygen levels and reduced survival rates. There is also some variation in the type of water that embryos are kept in, with protocols including fish or system water, methylene blue, or embryo water (also known as E2 or E3).

Upon completion of morphogenesis, early larval stage (3–13 dpf) rearing methods are no more standardized. Karen found no standard stocking density, a variety of food choices, and three different housing methods in use. The housing options from 4 dpf are either a recirculation system where the drip rate or water flow should be increased with growth or a static system where fry must be moved into a circulating system between 9 and 16 dpf. Stocking densities varied from 10 to 60 fry per liter, with the average around 15 to 20 fry per liter. The digestive tract has developed such that fry are capable of independent feeding at around 5 dpf. This is the point at which zebrafish use is regulated in the United Kingdom under the revised Animals (Scientific Procedures) Act 1986 (ASPA).^9^

At this stage, there are three feeding options in general use; a dry diet, live food, or a combination of these. Dry food provides a defined nutritional content that can be given with some precision if weighed out, and comes in a variety of sizes that should be increased as fry grow. However, there is no “standard” regarding the quantity to be given and the nutritional content of the different dry feeds also varies. Many protocols describe dry feed quantities in terms of “puffs per tank,” but of those that did provide a measure, the average volume was 0.03 mL (equivalent amount of dry food was not calculated). Although dry diet permits greater precision with respect to the nutrition provided, it can cause problems in static tanks if uneaten food accumulates at the bottom, and unlike live feed, it does not simulate natural feeding behaviors.

Live food is generally in the form of *Artemia*, *Paramecium*, or salt or fresh water rotifers. All of these contain a high protein content that promotes growth, provide a form of enrichment by stimulating natural foraging behaviors, and can be provided as a polyculture in which more than one species is given within a single tank. However, live food is variable in its nutritional content, can be difficult to culture, and is a potential source of contamination or water pollution. As with dried food, the quantity and size of live food should increase as zebrafish develop. The amount of live food given within the study varied considerably, with an average quantity ranging from 1 to 200 mL per tank. This variation in volume, coupled with the variable concentration of live feed, means that the actual amount of food available to the fish could not be calculated.

Some zebrafish facilities (e.g., UCL) are currently providing both, for example, fry may initially get dry food and move on to live food once they leave the nursery, or a combination of dry and live food may be given from the time when fish commence feeding with changes made to quantity and size according to fry growth and gape size. Of the centers currently providing both dry and live food, increments are commonly made around 10, 15, and 21–28 dpf, and at a fourth time point depending on growth. UCL has been conducting trials looking at the effect of five diets (*Paramecium*, fresh and saltwater rotifers, *Philodina*, and a control diet) on fry survival up to 15 dpf in static water housing. A second trial compared the combined effect on fry survival of three different diets (*Paramecium* and fresh and saltwater rotifers) and two different housing systems (static and recirculation). However, the results of these studies were not available for discussion at the meeting.

## The 3Rs in Large-Scale Colony Management

Dr. Elisabeth Busch-Nentwich (Wellcome Trust Sanger Institute) gave a presentation on the Zebrafish Mutation Project (ZMP^10^). The ZMP aims to create knockouts of all 26,000 protein coding genes in the zebrafish, with complete exon sequencing and multiallelic phenotype profiling of each mutant.

At the time of the meeting, around 32,000 alleles of around 14,000 protein coding genes had been identified, and around 2400 alleles had undergone morphological and molecular phenotyping. Initially the project used *N*-ethyl-*N*-nitrosourea (ENU) as a mutagen, but the CRISPR/Cas9 system is now used to knock out specific genes of interest. Phenotyping involves screening during the first 5 dpf and is proving effective with around 8% of mutated alleles having a detectable (morphological or behavioral) phenotype at this stage and around 12.5% of alleles being juvenile lethal. This approach also means fry carrying mutated alleles of potential interest can be selected to be raised to adulthood, thereby reducing the overall number of fish bred and maintained for experimental purposes.

Elisabeth also described the Wellcome Trust Sanger Institute's zebrafish facility, which houses around 77,000 adult fish and provides a large number of researchers with access to their stocks, either through frozen sperm samples or live F2 families. Every stage of zebrafish development is closely monitored and managed to enable lethality and welfare concerns to be minimized, while optimizing efficiency and capacity. Good husbandry is the top priority during the first 15 days postfertilization, and has proved to be essential in determining the quality of their fish stocks. This time is spent in the “nursery” where food and stocking density are the most critical factors with respect to minimizing unviable fry. In general, the largest losses are sustained within the first 30 days.

Like UCL, the Sanger Institute has trialed different feeding regimes in their “nursery”; on average, only 30% of mutated fry survived on a dry diet of ZM (Zebrafish Management Ltd., Winchester, UK) (ZM000 5–19 dpf, ZM100 20–34 dpf, ZM200 35–49 dpf) and 65% on a dry diet of Zeigler, with the most common defect observed in both cases being scoliosis. They also compared survival rates between fry fed *Paramecium* twice daily for the first 10 days, and then Zeigler dry food versus those given Zeigler dry food during the first 10 days. The results were 91% survival with *Paramecium* for the first 10 days versus 60% survival for those fed the dry diet only. As a result, current practice at the Sanger Institute is to keep 5–30 dpf fry at a stocking density of 50 fry per liter and feed them a diet of *Paramecium*. This enables the Institute to start with lower numbers of fry because their growth is quick and uniform, resulting in fish that are sexually mature at 10–12 weeks of age.

Elisabeth explained that these conditions require additional time and space requirements to culture the *Paramecium* and minimize the risk of introducing contaminated material into the breeding or experimental tanks and water system, but for them, the benefits outweigh these costs.

Breeding practices were discussed next, with 4- to 15-month-old zebrafish currently kept in mixed sex groups because separating females and males was found to decrease egg production. Results of “pair” versus “group” mating were compared, as well as variations in breeding tank size, shape, and structure. At the Sanger Institute, a mating tank with a sloping grid floor has been found to provide the most consistent and reliable mating trigger, with footage showing that spawning is stimulated as soon as the floor gradient is established. Some females do still become egg bound and these will be identified quickly and “squeezed” manually, under anesthesia, if required. The ZMP group also collects sperm to indefinitely archive mutant and transgenic lines.^11^ At present, they have around 24,000 alleles stored as frozen sperm samples in 10% *N*,*N*-dimethylacetamide in buffered sperm motility-inhibiting solution (DMA/BSMIS).

This buffer is easy to make and store, and in their hands, DMA has been found to be a more consistent cryoprotectant than methanol and powdered milk in Ringer's. The sperm samples are collected from 6- to 12-month-old males selected by size; bigger is considered better as larger males are able to produce around 4 μL of sperm, which can be split into eight aliquots. Sperm yield also increases threefold when males are kept separate from females for at least a week before collection. This keeps the number of males per line that need to be squeezed using abdominal massage to a minimum and also reduces the number of repeat procedures per male, which is capped at five times. Fresh sperm in BSMIS (without DMA) keeps for at least an hour on ice and only very little is needed for successful fresh *in vitro* fertilization (IVF), which is a good method to recover old lines where fish no longer mate naturally.

The ZMP prefers to use IVF for ZMP-mutagenized males because they are generally more delicate and mating can be stressful. For IVF using frozen sperm, a good breeding/squeezing schedule with productive females is essential, using around six females per sperm sample. Up to 60% of eggs are fertilized using frozen sperm, and Tupfel Long Fin females are very good, although they are prone to becoming egg bound.

Elisabeth finished her talk by discussing the importance of good record keeping in managing zebrafish colonies efficiently. This should not only minimize the number of fish being bred but also identify line-specific traits and potential welfare issues that may be ameliorated or avoided by refinements to housing, husbandry, or care practices. Good record keeping is a Home Office requirement for UK establishments, and there are many ways to achieve this. At the Sanger, they use a customized Filemaker Pro database for this purpose, but they also make good use of Google docs for some records, for example, nursery schedules, line records, RNA sample management, and genotyping. The ZMP makes much of this information freely available through its website, and other useful resources include Ensembl genome browser, ZFIN the zebrafish model organism database, ZIRC the Zebrafish International Resource Center, and EZRC the European Zebrafish Resource Center.

## Environmental Enrichment for Zebrafish

This topic was discussed by Dr. Lynne Sneddon (University of Liverpool). Zebrafish are the most common aquatic laboratory species and are viewed as a valuable experimental “model” that can be managed in a “high-throughput” manner. They are also a species for which enrichment is still not general practice.

Current opinions as to whether or not enrichment should be provided for zebrafish vary depending on the form of enrichment and the potential benefits for the animals. Some may consider that “enrichment is just aquarium decoration designed to make tanks look more pleasing to humans,” while others believe that “zebrafish need a complex, enriched environment for good health and welfare.” The latter attitude is accepted with respect to laboratory mice, and, while appropriate enrichment for fish species can vary widely according to habitat and life stage, consideration of the zebrafish' natural habitat^7^ suggests that efforts should be made to provide a good quality environment for the species.

There are also different approaches to enrichment, from providing “environmental heterogeneity” to “behavioral engineering,” be that social or sensory stimulation, new enclosures, nutrition, physical enrichment, or novel objects. A limited, but increasing number of research projects are evaluating different environmental enrichments and whether they can improve welfare and/or speed up recovery from stressful or painful procedures.

Lynne discussed the OECD guidelines for toxicology recommendation for environmental heterogeneity,^12^ which can be partly achieved for zebrafish through the addition of various lengths of inert glass rods (structural enrichment).^13^ In this case, there was an impact on the time taken to establish a social hierarchy, which involves aggressive behavior, in a barren versus a structurally enriched tank. The structured enrichment actually appeared to prolong aggression and the period taken to establish a social hierarchy, but when cortisol levels were checked as an indicator of stress, very little difference was found between the two environments.^13^

This study demonstrates that it is important to consider the effects of enrichments on behavior and how these may be experienced by the fish. More research is clearly needed into the effects of enrichment on zebrafish behavior, and preference tests may prove useful in identifying environmental enrichments that may improve welfare without increasing stress to unacceptable levels. For example, preference tests using zebrafish housed in barren tanks and with no prior exposure to enriched environments showed that 90% preferred the enriched quarter with stones and plants when given the choice.^14^ In another study, researchers investigated the effect on cortisol levels of different colors on the bottom of the tank, with the zebrafish showing a preference for dark colors (black, red, blue) or a barren tank over brighter colors such as green and white.^15^

**FIG. 1.**
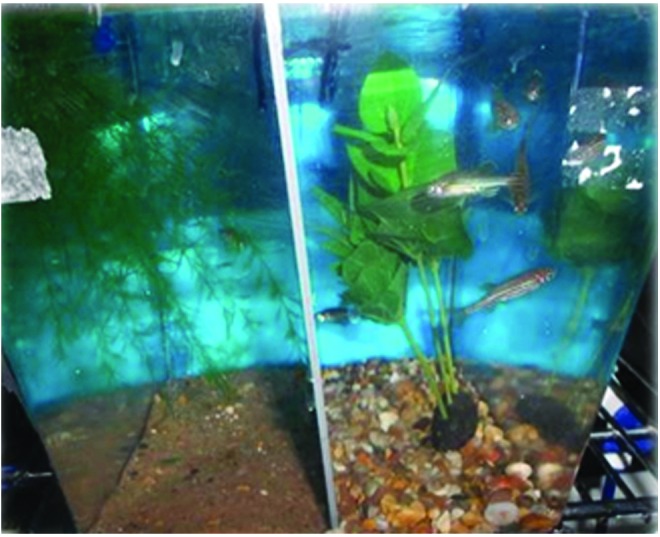
A zebrafish preference test. The fish chose to spend more time in the half of the tank with gravel and a submerged plant, rather than sand with a floating plant. Photo credit: P. Schroeder. Color images available online at www.liebertpub.com/zeb

Lynne also discussed work by Paul Schroeder, a researcher in her group, who “asked” fish what they wanted by pitting different resources against one another to determine whether there was an order of preference, and whether that varied with gender, competition, and dominance in pairs or in groups.^16^ Zebrafish were assessed either in male:female pairs or in groups of 4:4 male:female and their preferences were assessed for barren tanks versus those containing sand, gravel, or an image of both; submerged and rooted or floating plants providing overhead cover; or an airstone (Fig. 1). All of these “enrichments” were clearly preferred to a barren environment with the exception of the airstone. However, this research was conducted using AB zebrafish, so these results could be strain or genetic background specific. Paul went on to investigate further the preference for images of sand or gravel, with zebrafish displaying a clear preference for the section of the tank containing the image of gravel when compared with a barren tank, but no clear preference when the image of sand was used. This research suggests that zebrafish reared for 7 months in barren conditions have a preference for environmental heterogeneity, but it is less clear whether this promotes better welfare, whether fish will recover quicker from stress and pain, or if, in fact, there are any strain differences in these responses.^16^

To assess whether environmental enrichments might promote recovery and welfare, Lynne's group have housed two strains of 3-month-old zebrafish (AB and Leopard) in tanks containing a gravel image and floating plant for 4 months before testing their response to various stressors. These stressors were simulated predator presentation, air emersion, and fin clipping. Preliminary results suggested that the presence of enrichment had no impact on recovery, although there were substantial differences between the strains dependent upon the context, as behavioral responses were highly specific between the different stressor types. Enrichment, therefore, does not confound the quality of data collected.

Lynne's team is also researching the effect of environmental enrichments in rainbow trout (*Oncorhynchus mykiss*), golden sawfin goodeids (*Skiffia francesae*), guppies (*Poecilia reticulata*), and *Garra rufa* fish. So far, levels of anxiety seem to be lower across the range of fish species when housed in an enriched environment containing gravel and plants, but this type of enrichment appears to make very little difference to *G. rufa* fish for which higher stocking densities are more of a critical stressor. All of this work is limited to some degree because there are no known positive markers of fish welfare, or reduced stress, but as the amount of published research on fish environmental enrichment increases, a clear message is emerging that preference and the effect of environmental enrichments are very species and potentially strain specific.

## Poster Session

The last session of the day, hosted by Gregory Paull (University of Exeter), comprised several short talks from poster abstracts submitted by meeting participants.

Gregory opened the session by describing a project in his laboratory aiming to refine the common practice of pair housing a male and a female zebrafish 1:1 in a small (1–2 L) breeding chamber for a few hours, or up to a few days, for breeding and subsequent screening/genotyping. In large screening laboratories, many hundreds of pairs may be set up in this way on a daily basis. However, zebrafish can be very aggressive when kept at low densities, so this housing method could have consequences for both health and welfare. There have been anecdotal reports of declines in fish health, and even mortality, where pairs have been left together for too long (as little as a few hours can be detrimental) or wrongly size matched, but little or no research has been conducted into how to ensure good egg output, while maintaining fish welfare, when using this method.

Using behavior (measured by counting aggressive interactions between individuals, for example, chasing, repelling, sparring, and biting) as an indicator of stress, they found that both the size and the gender of the dominant individual affected the stress observed in the subordinate fish. Males were more likely to be dominant than females, but females with a body mass more than 10% greater than the male became the dominant individual, although they were significantly less aggressive than dominant males. Both levels of aggression and levels of subordinate stress were reduced further when the breeding chambers were “enriched” with a small piece of plastic plant. Therefore, pairing large females with smaller males in an enriched tank may be the best way to minimize the potential stresses of using monogamous breeding pairs in zebrafish colony management, although size matching alone (biased toward larger females) could be an equally important method.

Gregory felt that, while great strides had been made by industry in developing systems to efficiently house large numbers of fish, maximize growth to sexual maturity, and produce large numbers of embryos, energy should now be directed toward optimizing these common housing and handling practices through a better understanding of the interplay that exists between the social and physical environmental requirements of the fish and how these can impact on the well-being of the fish if one or both are changed.

Gregory went on to explain that, although zebrafish are a group-living species and have long been classified as a “community” species in the hobbyist trade, they still exhibit dominance hierarchies,^17^ which become amplified at lower densities (such as pair housing for mating), promoting aggression. In some zebrafish lines, this level of aggression can quickly, for example, after only a few hours, result in reduced welfare in the subordinate individual.^18^ This may even result in physical damage through constant biting or mouthing which, without intervention, can damage the mucus or epidermis of the subordinate individual resulting in secondary infections and in severe cases, mortality.^19^ This is clearly a welfare issue that needs to be investigated, given how routinely zebrafish are housed in pairs or at low density for specific practices.

The next presenters were Victoria King and Natalie Wren (National Institute for Medical Research [NIMR], now part of the Francis Crick Institute) who described a study comparing three different breeding regimes and their effect on zebrafish growth and fecundity. This compared three commercially available dry diets (ZM, Hikari, and TetraMin Baby) supplemented with *Artemia* (all diets), or a *Paramecium* and rotifer polyculture (ZM and Hikari only). All larvae were stocked at a density of 20 per tank with growth rates and survival rates comparable across all diets. However, those on the ZM diet grew 2% longer (total length 25 mm) than those fed the Hikari diet (total length 24.6 mm) and 14% longer than those on the TetraMin Baby diet (total length 21.6 mm).

Although there is no direct correlation between body condition and welfare, the better body condition we observed may be an indicator of good health, which is an essential component of good welfare. The ZM diet also proved to be the easiest to feed, leaving no oily residue in the water or tank.

Joe Higgins (NIMR, now Francis Crick Institute) described a study comparing how stocking density affects zebrafish growth rates. It has been shown that lower stocking densities result in faster growth, while higher stocking densities result in slower growth, but he also investigated whether either system had an impact on fish welfare. Wild-type Lon/AB strain zebrafish were used, fed on the NIMR standard feeding regime. After 2 months, fry were transferred into individual 3 L tanks at either 25 or 15 fry per tank, and fed the same standard amount in both stocking densities. Every 2 weeks, five fish were randomly picked from each tank and photographed so that they could be measured. Data collection and analysis was still underway, however preliminary results indicated no significant difference between the average lengths of fish kept at these two stocking densities.

## Audience Survey

A question and answer discussion session was held at the end of the meeting, using an electronic voting system (Turning Point) to enable individuals to answer questions anonymously as well as gather some data on a sample of current zebrafish facility practices. Some of these questions were preplanned to relate to the talk topics of the day and others were added during the session in response to the discussion.

The first question asked how space was allocated in the participants' facilities. Twenty-two percent said that users managed their own stock and space; 18% answered that users were assigned a standard number of tanks, or that space was allocated to whoever has the funding to cover the cost; 14% said that the decision lay with the facility manager; and 4% believed that space was randomly allocated. The remaining 25% of participants answered that space was allocated using a combination of all of these approaches. Each of these options had different pros and cons, including the impact on both colony management and the implementation of the 3Rs locally.

This session also identified where there was some consistency in practices and where there was more variation. For example, with respect to the medium in which facilities kept their embryos, methylene blue, fish/system water, and embryo water/E2/E3 were the most common (50%, 43% and 30%, respectively), but 4% kept their embryos in bleach solution. Answers were all anonymous so it was not possible to identify whether this was a special case, or for specific reasons, so discussion focused on why keeping in bleach solution was not routinely necessary.

The next question asked participants whether they removed debris from their nursery tanks. It was found that 38% always remove debris, 26% usually, 15% sometimes and 21% never. The following discussion clarified that the larger zebrafish facilities never removed debris from their nursery tanks, because in their experience survival rates were higher when disturbance was minimized.

Discussion then moved on to “nursery” diets with 17% using dry diet only and 17% feeding live diet only (*Paramecium*, *Artemia*, or saltwater rotifers), but the majority (74%) fed a combination of dry and live food, as discussed by presenters previously. All respondents varied the size and quantity of the food at each larval stage, with the majority (65%) doing so at all stages and the remaining 35% for some stages. Participants were more split when asked how often they fed larval forms/fry. Most (57%) said thrice a day and 20% said it varied, with the remaining respondents answering four times a day (18%) or twice a day (5%).

Another practice where there was more consistency, at least between the meeting participants, was the housing of males and females. Sixty-seven percent always housed both sexes together in groups and the remaining 33% varied between groups and pairs. There was more variation in stocking densities per liter, with 58% stocking at 5 fish per liter; 14% stocking at 6–10; 6% stocking at less than 4 or more than 10; and the remaining 17% varying their stocking density. There was also variation in the average age at which fish within facilities are reaching sexual maturity. This is obviously strain dependent, but 50% responded that fish were mature at 3 months, 35% at 4 months, 12% at 5 months, and the remaining 6% at less than 2 months.

Discussion included how to balance the benefits to researchers of reducing the time taken to reach sexual maturity against the potential adverse consequences for the health and welfare, or lifetime experience, of the fish. Interestingly, there was less variation in the age at which facilities stopped using fish for breeding, with 44% doing so at 12–18 months and the remaining responses equally divided between over 18 months and “it varies.”

Regarding access to cryopreservation services, 52% of respondents had no access, while 45% had “in-house” expertise and 3% used external services. When asked how often facilities allowed either male or female fish to be “squeezed” over their lifetime, 11% of respondents said only once, 37% said two to four times, and the remaining 53% said five or more times.

Participants were asked their views on environmental enrichment, as a follow on from Lynne's talk. Forty-two percent had the view that it was not always appropriate, 31% said it was a “good to have,” 14% were not sure, 11% thought it was essential, and 3% thought it was not appropriate. So, clearly, there is a need for more research evaluating the health, welfare, and scientific benefits of enrichment, to help make informed decisions regarding what to provide, at which stages, and within which projects. On a more positive note, 100% of participants said they always performed visual checks on the health and welfare of their zebrafish. They also routinely tracked and monitored aspects of behavior, fecundity, and health.

## Conclusion

All of the talks and discussion on the day demonstrated a strong interest in defining good practice for breeding, housing, husbandry, and care for laboratory zebrafish, and helped to highlight areas that especially needed to be standardized. All of this would improve zebrafish health and welfare and also likely lead to scientific benefits, due to greater standardization of lines and husbandry protocols.

The RSPCA published a global review of current practices in 2010,^7^ but further efforts are needed to share information on good approaches to zebrafish breeding, housing, husbandry, and care. More research is also needed, to define good practice; for example, more indicators of stress and poor welfare—and of good welfare—should be evaluated for zebrafish. This would help to explore important issues, for example, which stocking densities and environmental enrichment protocols are most appropriate in a given situation, and could also help to resolve dilemmas such as rapid maturation versus adverse welfare consequences for individual fish.

The work of the TTWG has now ceased, but we hope that this meeting report stimulates further discussion, liaison, and meetings between those who use and care for laboratory zebrafish, with the aim of fully implementing the 3Rs and actively working to improve their welfare.
